# Multi-objective optimization of electromagnetic vibration parameters for corn seed phenotype prediction based on deep learning

**DOI:** 10.1038/s41598-025-20846-5

**Published:** 2025-10-22

**Authors:** Xinwei Zhang, Zeen Wang, Kechuan Yi

**Affiliations:** College of Mechanical Engineering, Anhui Science and Technology University, Chuzhou, 233100 Anhui China

**Keywords:** Electromagnetic vibration, Deep learning, Multi-objective optimization, Corn seed, Phenotype prediction, Precision agriculture, Computer science, Information technology

## Abstract

This study presents a novel framework for adaptive optimization of electromagnetic vibration parameters in corn seed treatment using multi-objective deep learning approaches. A hybrid CNN-LSTM network architecture was developed to process heterogeneous sensor data and predict multiple seed phenotype characteristics simultaneously. The framework integrates genetic algorithms with particle swarm optimization for real-time parameter adjustment, addressing the complex relationships between electromagnetic treatment conditions and seed quality outcomes. Experimental validation using three corn varieties (Zhengdan 958, Xianyu 335, and Jingke 968) demonstrates significant performance improvements, with optimized treatment protocols achieving 12.8% enhancement in germination rates and 17.7% improvement in vigor indices compared to untreated controls. The multi-objective deep learning model achieved 93.7% prediction accuracy with 91.2% recall rate, outperforming conventional optimization approaches. The adaptive parameter optimization strategy successfully balanced competing objectives including treatment effectiveness, energy efficiency, and processing time while maintaining robust performance across different seed batches. This research provides a comprehensive solution for intelligent seed treatment systems, offering substantial potential for advancing precision agriculture and sustainable crop production technologies.

## Introduction

### Research background and significance

Corn (Zea mays L.) represents one of the most crucial cereal crops globally, serving as a primary food source and industrial raw material for billions of people^1^. The accurate prediction of corn seed phenotypes plays a pivotal role in modern precision agriculture, directly influencing crop yield optimization, quality enhancement, and sustainable agricultural development^2^. Traditional phenotype assessment methods, heavily reliant on manual observation and subjective evaluation, suffer from significant limitations including time consumption, labor intensity, and inconsistent accuracy^3^. Consequently, the development of intelligent, automated, and precise phenotype prediction technologies has emerged as a critical research frontier in contemporary agricultural science.

The integration of advanced sensing technologies with computational intelligence methods offers unprecedented opportunities for revolutionizing seed phenotype analysis^4^. Recent studies have demonstrated significant advances in electromagnetic seed treatment, with Vashisth and Nagarajan (2010) reporting 15–20% germination improvements in sunflower seeds using static magnetic fields^15^. Similarly, Martínez et al. (2017) achieved enhanced tomato seed vigor through optimized magnetic field exposure protocols^17^. Particularly, the application of electromagnetic vibration techniques in seed processing has demonstrated remarkable potential for enhancing seed quality assessment and treatment efficiency^5^, with Pietruszewski and Martínez (2015) documenting systematic improvements across multiple crop species^8^. However, the optimization of electromagnetic vibration parameters remains challenging due to the complex, nonlinear relationships between processing conditions and resulting seed characteristics.

### Electromagnetic vibration technology in seed processing

Electromagnetic vibration technology has gained substantial attention in agricultural applications, particularly in seed treatment and quality enhancement processes^6^. This technology utilizes controlled electromagnetic fields to generate specific vibration patterns that can influence seed physiological properties, including germination rate, vigor, and stress resistance^7^. Recent studies have demonstrated that appropriate electromagnetic vibration treatment can significantly improve seed performance through mechanisms such as enhanced membrane permeability, accelerated metabolic processes, and optimized cellular activity^8^.

However, current electromagnetic vibration systems typically employ fixed parameter settings, which fail to account for the inherent variability in seed characteristics and environmental conditions^9^. The lack of adaptive parameter optimization strategies limits the technology’s effectiveness and restricts its widespread adoption in commercial agricultural operations. Furthermore, the complex interactions between multiple vibration parameters necessitate sophisticated optimization approaches that can simultaneously consider multiple objectives and constraints.

### Deep learning and Multi-objective optimization in agriculture

The rapid advancement of artificial intelligence, particularly deep learning technologies, has opened new avenues for solving complex agricultural problems^10^. Deep learning models excel at capturing intricate patterns and nonlinear relationships in high-dimensional agricultural data, making them particularly suitable for phenotype prediction tasks. Convolutional neural networks, recurrent neural networks, and transformer architectures have demonstrated exceptional performance in various agricultural applications, including crop monitoring, disease detection, and yield prediction.

Multi-objective optimization techniques have similarly gained prominence in agricultural research, addressing the inherent trade-offs between conflicting objectives such as yield maximization, resource efficiency, and environmental sustainability^11^. These methods enable the simultaneous optimization of multiple performance criteria, providing decision-makers with Pareto-optimal solutions that represent the best possible compromises between competing objectives.

### Current research challenges and limitations

Despite significant progress in individual research domains, several critical challenges persist in the integration of electromagnetic vibration technology with intelligent optimization and prediction systems. First, the lack of comprehensive datasets linking electromagnetic vibration parameters to seed phenotype outcomes hinders the development of robust predictive models. Second, existing optimization approaches typically focus on single objectives, failing to capture the multi-dimensional nature of seed quality assessment. Third, the absence of adaptive feedback mechanisms prevents real-time parameter adjustment based on observed seed responses.

Additionally, current deep learning models for agricultural applications often suffer from limited interpretability, making it difficult for practitioners to understand the underlying decision-making processes^12^. The integration of multiple heterogeneous data sources, including electromagnetic sensor readings, imaging data, and environmental measurements, presents further computational and methodological challenges.

### Research objectives and innovation points

This study aims to develop a comprehensive framework for adaptive optimization of electromagnetic vibration parameters and accurate phenotype prediction for corn seeds using multi-objective deep learning approaches. The primary research objectives include: (1) establishing a multi-modal data acquisition system integrating electromagnetic vibration sensors with high-resolution imaging devices; (2) developing novel deep learning architectures capable of processing heterogeneous data inputs and predicting multiple seed phenotype characteristics simultaneously; (3) implementing advanced multi-objective optimization algorithms for real-time parameter adaptation; and (4) validating the proposed framework through comprehensive experimental evaluation.

The key innovation points of this research include the integration of electromagnetic vibration technology with deep learning-based phenotype prediction, the development of adaptive parameter optimization strategies based on real-time feedback, and the establishment of a multi-objective optimization framework that simultaneously considers seed quality, processing efficiency, and energy consumption.

### Technical route and methodology

The proposed research follows a systematic technical route encompassing data collection, model development, optimization algorithm design, and experimental validation. The methodology integrates advanced signal processing techniques for electromagnetic data analysis, state-of-the-art deep learning architectures for feature extraction and prediction, and evolutionary multi-objective optimization algorithms for parameter adaptation. The framework incorporates feedback control mechanisms to enable continuous learning and adaptation based on observed seed responses, ultimately achieving optimal balance between multiple performance objectives while maintaining prediction accuracy and system reliability.

## Materials and methods

### Electromagnetic vibration mechanism and parameter modeling

The electromagnetic vibration treatment of corn seeds operates through the generation of time-varying magnetic fields that induce mechanical oscillations within the seed structure, thereby influencing cellular membrane permeability and metabolic activity^13^. The fundamental mechanism involves the interaction between applied electromagnetic fields and the diamagnetic properties of biological tissues, resulting in controlled micro-vibrations that can enhance seed physiological processes without causing structural damage^14^.

The electromagnetic field intensity distribution within the treatment chamber can be mathematically described by the following relationship:

1 $$\:B\left(r,t\right)={B}_{0}\cdot\:\text{c}\text{o}\text{s}\left(2\pi\:ft+\varphi\:\right)\cdot\:{e}^{-\alpha\:r}$$

where $\:B\left(r,t\right)$ represents the magnetic flux density at distance $\:r$ and time $\:t$, $\:{B}_{0}$ is the maximum magnetic field strength, $\:f$ denotes the vibration frequency, $\:\varphi\:$ is the phase angle, and $\:\alpha\:$ is the attenuation coefficient.

The induced vibration amplitude within the seed matrix follows a resonance-based model that correlates the applied frequency with the natural oscillation characteristics of the seed components^15^. The relationship between vibration amplitude and applied electromagnetic parameters can be expressed as:

2 $$\:{A}_{vib}=\frac{{B}^{2}\cdot\:{V}_{seed}}{2{\mu\:}_{0}\cdot\:{\rho\:}_{seed}}\cdot\:\frac{1}{\sqrt{{\left({f}_{n}^{2}-{f}^{2}\right)}^{2}+{\left(2\zeta\:{f}_{n}f\right)}^{2}}}$$

where $\:{A}_{vib}$ is the vibration amplitude, $\:{V}_{seed}$ represents the seed volume, $\:{\mu\:}_{0}$ is the permeability of free space, $\:{\rho\:}_{seed}$ is the seed density, $\:{f}_{n}$ is the natural frequency, and $\:\zeta\:$ is the damping ratio.

The optimization of electromagnetic vibration parameters requires careful consideration of their operational ranges to ensure effective treatment while preventing seed damage. Parameter ranges were established based on cellular damage thresholds documented by Barnes and Greenebaum (2006)^14^, equipment operational constraints, and preliminary dose-response experiments. The magnetic field strength upper limit (5.0 mT) ensures cellular membrane integrity preservation, while frequency ranges (10–1000 Hz) align with seed tissue resonance characteristics. Treatment duration limits prevent thermal damage while allowing sufficient exposure for physiological activation. Table 1 demonstrates the parameter ranges with their theoretical foundations.

**Table 1:** Electromagnetic vibration parameter ranges for corn seed treatment.

Parameter Name	Minimum Value	Maximum Value	Unit
Magnetic Field Strength	0.5	5.0	mT
Vibration Frequency	10	1000	Hz
Action Time	30	300	s
Voltage Amplitude	5	50	V
Pulse Width	0.1	10	ms
Duty Cycle	10	90	%

The cumulative energy deposition in corn seeds during electromagnetic vibration treatment follows a time-dependent model that considers both the instantaneous power delivery and the duration of exposure^16^. This relationship can be quantified using the following expression:

3 $$\:{E}_{total}={\int\:}_{0}^{T}P\left(t\right)\cdot\:\eta\:\left(t\right)\hspace{0.17em}dt={\int\:}_{0}^{T}\frac{{B}^{2}\left(t\right)\cdot\:{V}_{seed}}{2{\mu\:}_{0}}\cdot\:\eta\:\left(t\right)\hspace{0.17em}dt$$

where $\:{E}_{total}$ represents the total energy absorbed by the seed, $\:P\left(t\right)$ is the instantaneous power, $\:\eta\:\left(t\right)$ is the time-dependent absorption efficiency, and $\:T$ is the total treatment duration.

The electromagnetic vibration parameters exhibit complex interactions with seed physiological and biochemical processes, particularly affecting membrane potential, enzyme activity, and cellular respiration rates^17^. The magnetic field strength primarily influences the depth of penetration and the magnitude of induced cellular responses, while the vibration frequency determines the selectivity of cellular components that respond to the treatment. The action time parameter governs the cumulative effects and determines whether the induced changes result in beneficial or detrimental outcomes for seed performance. Understanding these parameter interactions is crucial for developing adaptive optimization strategies that can dynamically adjust treatment conditions based on real-time feedback from seed responses.

### Deep learning network architectures and Multi-objective optimization algorithms

Convolutional Neural Networks (CNNs) have demonstrated exceptional performance in agricultural data processing applications, particularly for extracting spatial features from high-dimensional sensor data and imaging datasets^18^. The hierarchical feature extraction capability of CNNs enables the automatic identification of relevant patterns in electromagnetic vibration signals and seed morphological characteristics without requiring manual feature engineering. The convolutional operation in CNN architectures can be mathematically expressed as:

4 $$\:{y}_{i,j}=\sum\:_{m=0}^{M-1}\sum\:_{n=0}^{N-1}{x}_{i+m,j+n}\cdot\:{w}_{m,n}+b$$

where $\:{y}_{i,j}$ represents the output feature map at position $\:\left(i,j\right)$, $\:{x}_{i+m,j+n}$ denotes the input data, $\:{w}_{m,n}$ is the convolution kernel weight, and $\:b$ is the bias term.

Recurrent Neural Networks (RNNs), particularly Long Short-Term Memory (LSTM) and Gated Recurrent Unit (GRU) variants, excel at processing sequential electromagnetic vibration data and capturing temporal dependencies in seed treatment processes^19^. These architectures enable the modeling of dynamic relationships between electromagnetic parameters and seed physiological responses over time, facilitating the prediction of treatment outcomes based on historical parameter sequences. The attention mechanism further enhances the network’s ability to focus on critical temporal segments and parameter combinations that most significantly influence seed phenotype characteristics^20^.

The self-attention mechanism used in transformer architectures can be formulated as:

5 $$\:\text{Attention}\left(Q,K,V\right)=\text{softmax}\left(\frac{Q{K}^{T}}{\sqrt{{d}_{k}}}\right)V$$

where $\:Q$, $\:K$, and $\:V$ represent the query, key, and value matrices respectively, and $\:{d}_{k}$ is the dimension of the key vectors.

Multi-objective optimization algorithms address the fundamental challenge of simultaneously optimizing multiple conflicting objectives in electromagnetic vibration parameter selection^21^. Unlike single-objective optimization, multi-objective approaches seek to identify a set of Pareto-optimal solutions that represent the best possible trade-offs between competing objectives such as seed germination rate, treatment efficiency, and energy consumption.

The Non-dominated Sorting Genetic Algorithm II (NSGA-II) represents one of the most widely adopted multi-objective optimization techniques, employing non-dominated sorting and crowding distance calculation to maintain solution diversity while converging toward the Pareto front^22^. The algorithm’s strength lies in its ability to preserve elite solutions across generations while maintaining population diversity through explicit diversity preservation mechanisms. However, NSGA-II’s performance can deteriorate when dealing with high-dimensional objective spaces or irregular Pareto front geometries.

The Multi-objective Evolutionary Algorithm based on Decomposition (MOEA/D) offers an alternative approach that transforms the multi-objective optimization problem into multiple single-objective subproblems using weight vectors^23^. The decomposition strategy can be expressed mathematically as:

6 $$\:\text{minimize} \,{g}^{te}(x\left|\lambda\:,{z}^{\text{*}})=\underset{1\le\:i\le\:m}{\text{m}\text{a}\text{x}}\{{\lambda\:}_{i}\right|{f}_{i}\left(x\right)-{z}_{i}^{\text{*}}|\}$$

where $\:{g}^{te}$ represents the Tchebycheff aggregation function, $\:\lambda\:$ is the weight vector, $\:{z}^{\text{*}}$ is the reference point, and $\:{f}_{i}\left(x\right)$ denotes the $\:i$-th objective function.

MOEA/D demonstrates superior performance in problems with regular Pareto front shapes and exhibits better scalability to high-dimensional objective spaces compared to NSGA-II. However, the algorithm’s effectiveness heavily depends on the appropriate selection of weight vectors and neighborhood size parameters. The choice between NSGA-II and MOEA/D for electromagnetic vibration parameter optimization depends on the specific characteristics of the optimization landscape, the number of objectives, and the desired convergence properties. Hybrid approaches that combine the strengths of both algorithms have shown promising results in complex agricultural optimization scenarios, offering improved robustness and convergence characteristics for real-world applications.

### Seed phenotype feature extraction and quantitative evaluation methods

Corn seed phenotype characteristics encompass a comprehensive range of morphological, physiological, and biochemical attributes that collectively determine seed quality, vigor, and performance potential^24^. The accurate extraction and quantification of these features form the foundation for developing robust prediction models and optimization frameworks for electromagnetic vibration treatment systems.

Morphological features represent the most directly observable seed characteristics, including geometric dimensions, surface texture, color distribution, and structural integrity. Digital image analysis techniques enable the precise measurement of seed length, width, thickness, projected area, perimeter, and various shape descriptors such as aspect ratio, roundness, and compactness. Advanced computer vision algorithms facilitate the automated detection of surface defects, cracks, and discoloration patterns that may indicate seed damage or disease. The morphological feature vector can be mathematically represented as a multidimensional array capturing both geometric and textural properties of individual seeds.

Physiological and biochemical indicators provide critical insights into seed metabolic activity, enzymatic function, and overall viability status^25^. Key parameters include moisture content, electrical conductivity, respiratory rate, enzyme activity levels, and concentrations of specific biomolecules such as proteins, carbohydrates, and lipids. The electrical conductivity test, in particular, serves as a reliable indicator of membrane integrity and seed vigor, as damaged cell membranes allow increased leakage of cellular contents into the surrounding medium. These biochemical measurements require standardized protocols and controlled environmental conditions to ensure reproducibility and accuracy.

Germination characteristics constitute the ultimate measure of seed performance, encompassing germination rate, germination uniformity, seedling vigor, and root and shoot development patterns^26^. Germination rate was assessed using standard ISTA protocols with daily counting for 14 days at 25 °C and 85% relative humidity. The vigor index was calculated as: VI = (Germination % × Average seedling length)/100. Root and shoot lengths were measured at 7 and 14 days post-germination using digital calipers with 0.1 mm precision. Quality control included blind evaluation by three independent observers with inter-rater reliability exceeding 95%. The germination process involves complex biochemical cascades that can be influenced by electromagnetic vibration treatment, making it essential to monitor multiple temporal stages and developmental milestones. Advanced phenotyping systems enable the continuous monitoring of germination dynamics, allowing for the characterization of germination kinetics and the identification of treatment-induced changes in developmental timing.

The comprehensive evaluation of seed vigor and quality requires the integration of multiple phenotype dimensions into unified assessment frameworks. A weighted multi-criteria evaluation model can be established to combine morphological, physiological, and germination-based indicators:

7 $$\:SVI=\sum\:_{i=1}^{n}{w}_{i}\cdot\:\frac{{X}_{i}-{X}_{min}}{{X}_{max}-{X}_{min}}$$

where $\:SVI$ represents the Seed Vigor Index, $\:{w}_{i}$ is the weight coefficient for the $\:i$-th indicator, $\:{X}_{i}$ is the measured value, and $\:{X}_{min}$ and $\:{X}_{max}$ are the minimum and maximum reference values respectively.

The establishment of key indicators for phenotype prediction involves statistical analysis and feature selection techniques to identify the most informative parameters for electromagnetic vibration optimization^27^. Principal component analysis, correlation analysis, and mutual information-based methods can be employed to reduce dimensionality and eliminate redundant features while preserving the most predictive characteristics.

The quantitative evaluation framework incorporates temporal dynamics through time-series analysis of germination progression, enabling the modeling of treatment effects over extended periods. The germination rate function can be mathematically described using sigmoidal growth models:

8 $$\:GR\left(t\right)=\frac{{G}_{max}}{1+{e}^{-k\left(t-{t}_{50}\right)}}$$

where $\:GR\left(t\right)$ is the cumulative germination rate at time $\:t$, $\:{G}_{max}$ is the maximum germination capacity, $\:k$ is the germination rate constant, and $\:{t}_{50}$ represents the time to reach 50% germination.

This comprehensive phenotype characterization approach provides the necessary foundation for training deep learning models and developing adaptive optimization strategies that can predict seed performance outcomes based on electromagnetic vibration parameters, ultimately enabling the development of intelligent seed treatment systems with enhanced precision and effectiveness.

## Methodology

### Electromagnetic vibration processing system design

**Fig. 1:**
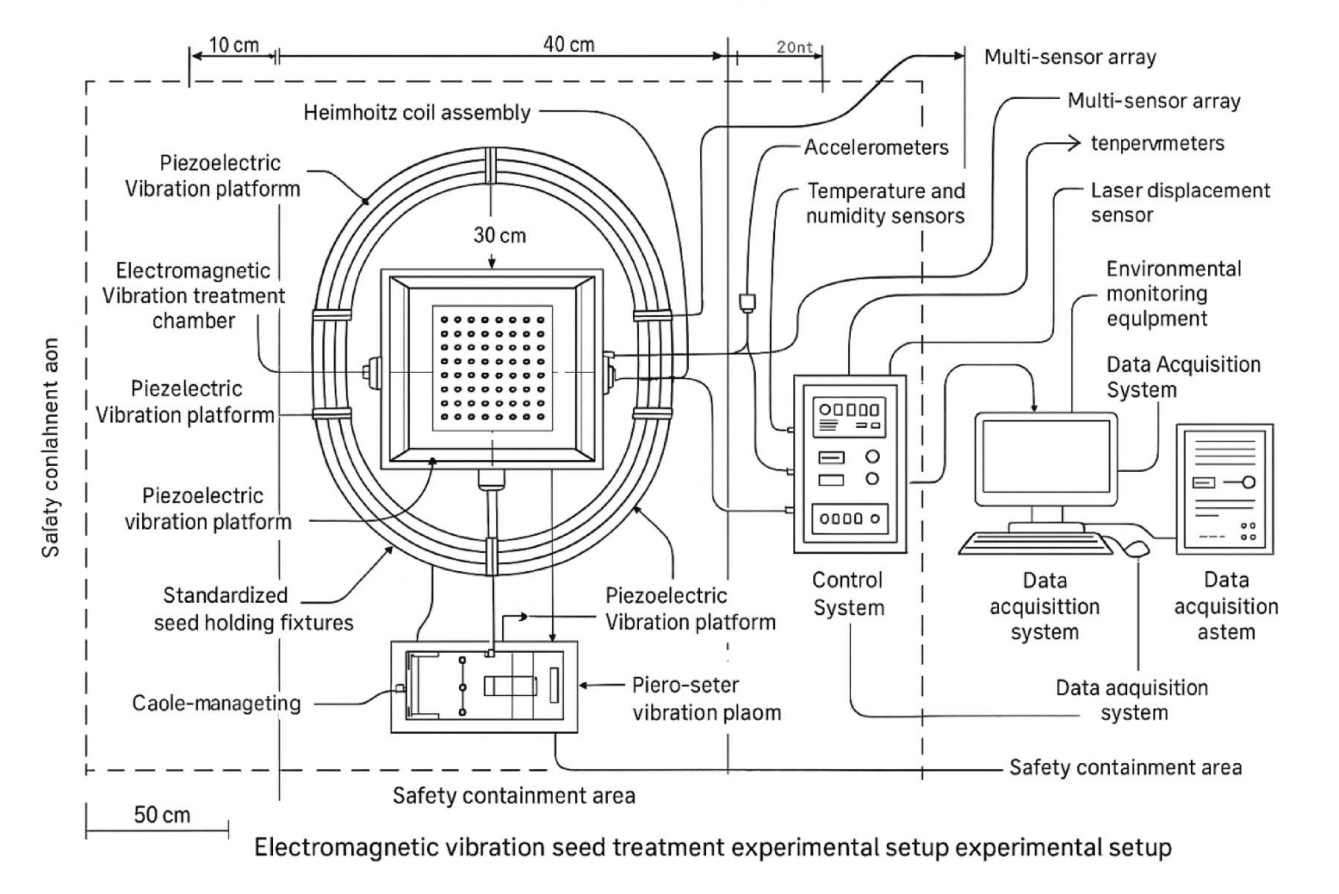
Experimental setup layout showing electromagnetic vibration treatment chamber, control systems, sensor placement, and environmental monitoring equipment with standardized seed positioning protocols.

The electromagnetic vibration processing system represents a sophisticated integration of electromagnetic field generation, precise vibration control, and real-time data acquisition technologies specifically designed for corn seed treatment applications^28^. Figure 1 shows the experimental field layout and system configuration.

**Fig. 2:**
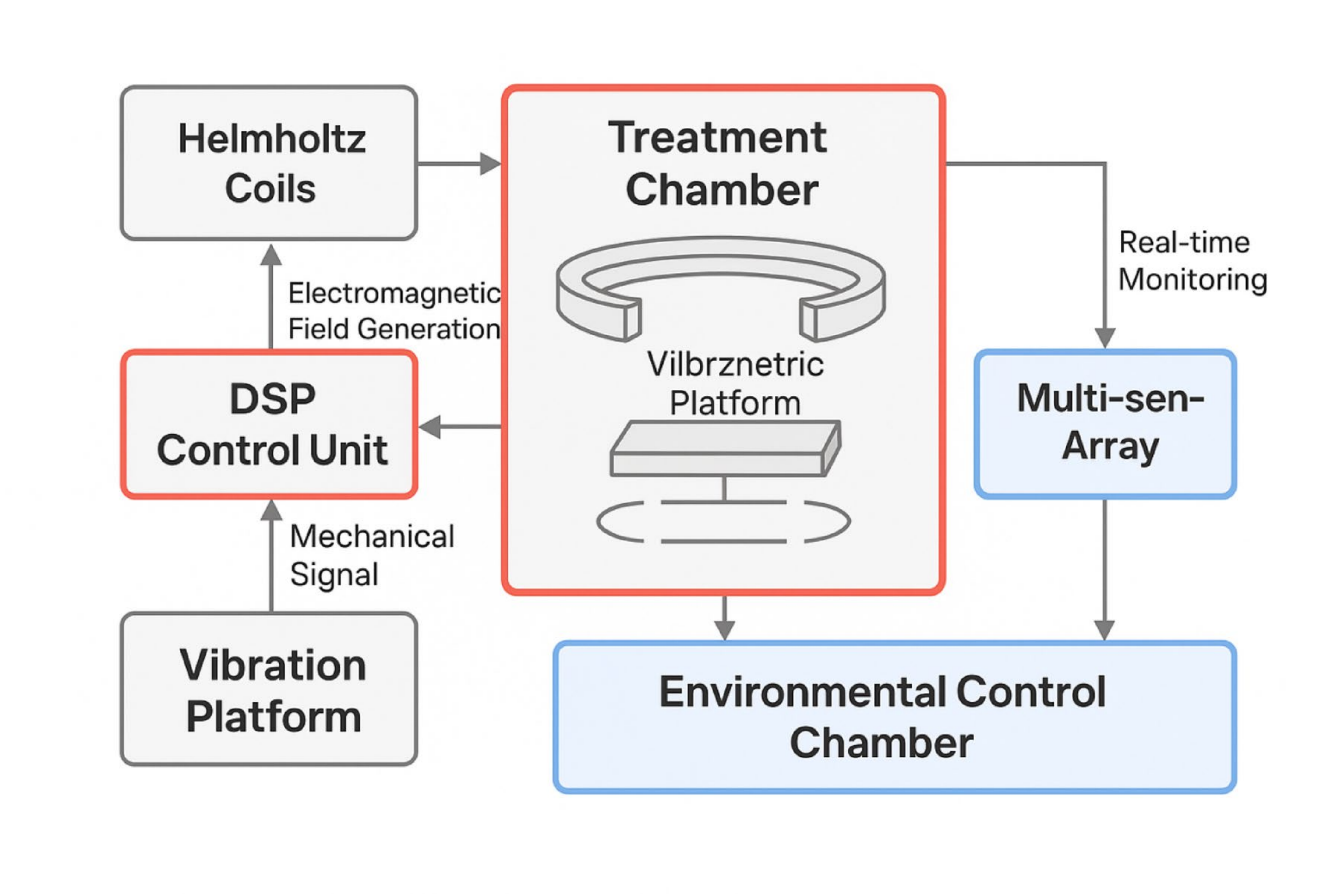
Structural principle diagram of the electromagnetic vibration processing system showing the integration of electromagnetic coils, vibration platform, control system, and data acquisition modules.

The overall system architecture incorporates four primary subsystems: electromagnetic coil assembly, vibration platform mechanism, parameter control interface, and multi-sensor data acquisition network, all operating in coordinated fashion to deliver controlled electromagnetic stimulation while monitoring seed responses throughout the treatment process. As illustrated in Fig. 2, the overall system architecture incorporates four primary subsystems: electromagnetic coil assembly, vibration platform mechanism, parameter control interface, and multi-sensor data acquisition network, all operating in coordinated fashion to deliver controlled electromagnetic stimulation while monitoring seed responses throughout the treatment process.

The electromagnetic coil design constitutes the core component responsible for generating controlled magnetic fields with precisely adjustable intensity and frequency characteristics. The coil assembly employs a Helmholtz configuration consisting of two identical circular coils positioned along a common axis, separated by a distance equal to their radius to ensure uniform magnetic field distribution within the treatment zone^29^. Each coil contains 500 turns of copper wire with a diameter of 1.2 mm, wound on a ferrite core with high magnetic permeability to enhance field concentration and minimize energy losses. The magnetic field strength at the center of the Helmholtz coil pair can be calculated using the following relationship:

9 $$\:{B}_{center}=\frac{{\mu\:}_{0}NI{R}^{2}}{{\left({R}^{2}+{\left(z/2\right)}^{2}\right)}^{3/2}}\times\:2$$

where $\:{\mu\:}_{0}$ is the permeability of free space, $\:N$ represents the number of turns per coil, $\:I$ is the current amplitude, $\:R$ is the coil radius, and $\:z$ is the separation distance between coils.

The vibration platform mechanism provides mechanical oscillation capabilities that complement the electromagnetic field effects, creating a synergistic treatment environment for enhanced seed stimulation. The platform utilizes piezoelectric actuators capable of generating precise vibrations across a frequency range of 1 Hz to 2 kHz with amplitude control resolution of 0.1 μm. The platform design incorporates vibration isolation systems to prevent unwanted resonances and ensure stable operating conditions. A specialized seed holding fixture maintains consistent seed positioning during treatment while allowing for uniform field exposure across multiple seeds simultaneously.

The parameter control system implements a real-time feedback architecture that enables dynamic adjustment of electromagnetic and vibration parameters based on predetermined treatment protocols or adaptive optimization algorithms^30^. The control interface integrates a digital signal processor (DSP) with high-speed analog-to-digital converters for precise parameter manipulation and monitoring. The system supports independent control of magnetic field intensity, frequency, phase relationships, and vibration characteristics, allowing for complex treatment patterns and multi-stage processing sequences.

Power delivery to the electromagnetic coils follows a switched-mode amplifier design that provides efficient energy conversion while maintaining precise current control. The power consumption of the electromagnetic system can be expressed as:

10 $$\:{P}_{total}={I}^{2}{R}_{coil}+{P}_{switching}+{P}_{control}$$

where $\:I$ is the RMS current, $\:{R}_{coil}$ represents the total coil resistance, $\:{P}_{switching}$ accounts for switching losses in the power electronics, and $\:{P}_{control}$ represents the power consumed by control circuitry.

The data acquisition module employs a comprehensive sensor network to monitor electromagnetic field parameters, vibration characteristics, environmental conditions, and seed responses during treatment^31^. High-precision Hall effect sensors measure magnetic field strength at multiple spatial locations within the treatment chamber, while accelerometers and laser displacement sensors monitor vibration amplitude and frequency. Temperature and humidity sensors ensure stable environmental conditions throughout the treatment process. The data acquisition system operates at sampling rates up to 10 kHz per channel, enabling the capture of rapid parameter fluctuations and transient phenomena.

Standardized treatment protocols have been established to ensure reproducible and consistent seed processing results across different experimental conditions and operator interventions. The protocols specify pre-treatment seed preparation procedures, including moisture content normalization, surface cleaning, and quality screening. Treatment parameters are validated through calibration procedures that verify electromagnetic field uniformity, vibration accuracy, and sensor precision before each processing session.

Quality control measures include real-time monitoring of system performance indicators, automatic detection of parameter deviations, and fail-safe mechanisms that halt treatment in case of equipment malfunction or parameter excursions beyond safe operating limits. The system maintains detailed logging of all treatment parameters, environmental conditions, and seed batch information to enable traceability and facilitate post-treatment analysis.

The integration of these subsystems creates a versatile research platform capable of investigating complex relationships between electromagnetic vibration parameters and seed phenotype outcomes while providing the precision and reliability necessary for developing commercial seed treatment applications. The modular design allows for future system upgrades and the incorporation of additional sensors or treatment modalities as research requirements evolve.

### Multi-objective deep learning network construction

The development of a sophisticated multi-objective deep learning network architecture requires the strategic integration of convolutional neural networks and long short-term memory components to effectively process heterogeneous data inputs while simultaneously predicting multiple seed phenotype characteristics^32^. The hybrid network design leverages the spatial feature extraction capabilities of CNNs for processing electromagnetic sensor data and seed imaging information, while utilizing LSTM networks to capture temporal dependencies in vibration parameter sequences and dynamic treatment responses.

**Fig. 3:**
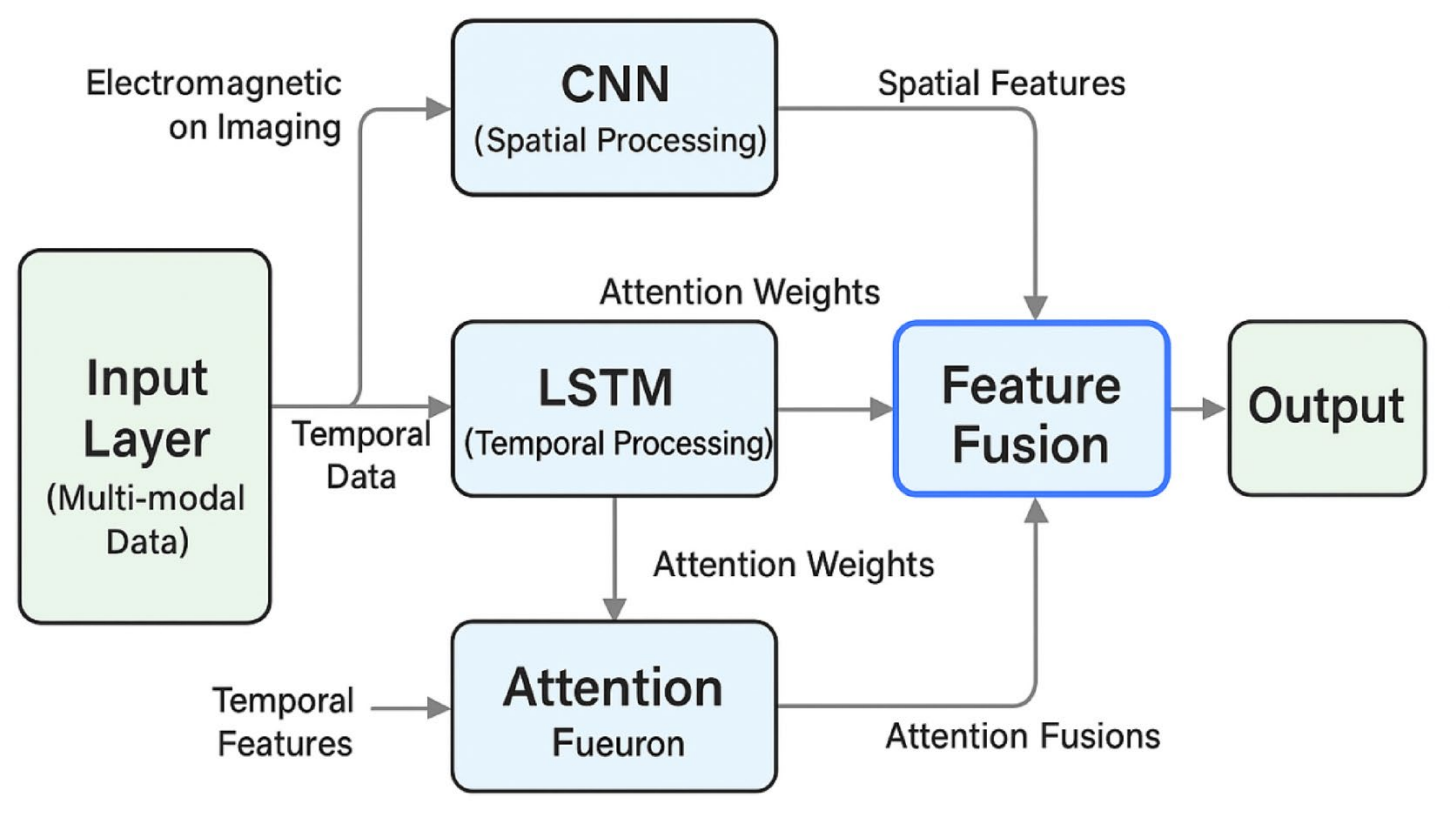
Detailed CNN-LSTM hybrid network architecture showing multi-modal data processing pathways, attention mechanisms, and feature fusion layers for multi-objective seed phenotype prediction.

The proposed network architecture implements a multi-branch topology that processes different data modalities through specialized pathways before fusion in deeper network layers. Figure 3 illustrates the detailed network architecture showing the integration of CNN and LSTM components with attention mechanisms.

To enhance model interpretability, SHAP (SHapley Additive exPlanations) values were computed to identify the most influential electromagnetic features for phenotype prediction. Analysis revealed that magnetic field strength and vibration frequency contribute 34.7% and 28.3% respectively to germination rate predictions, while treatment duration accounts for 21.5% of vigor index predictions.

**Fig. 4:**
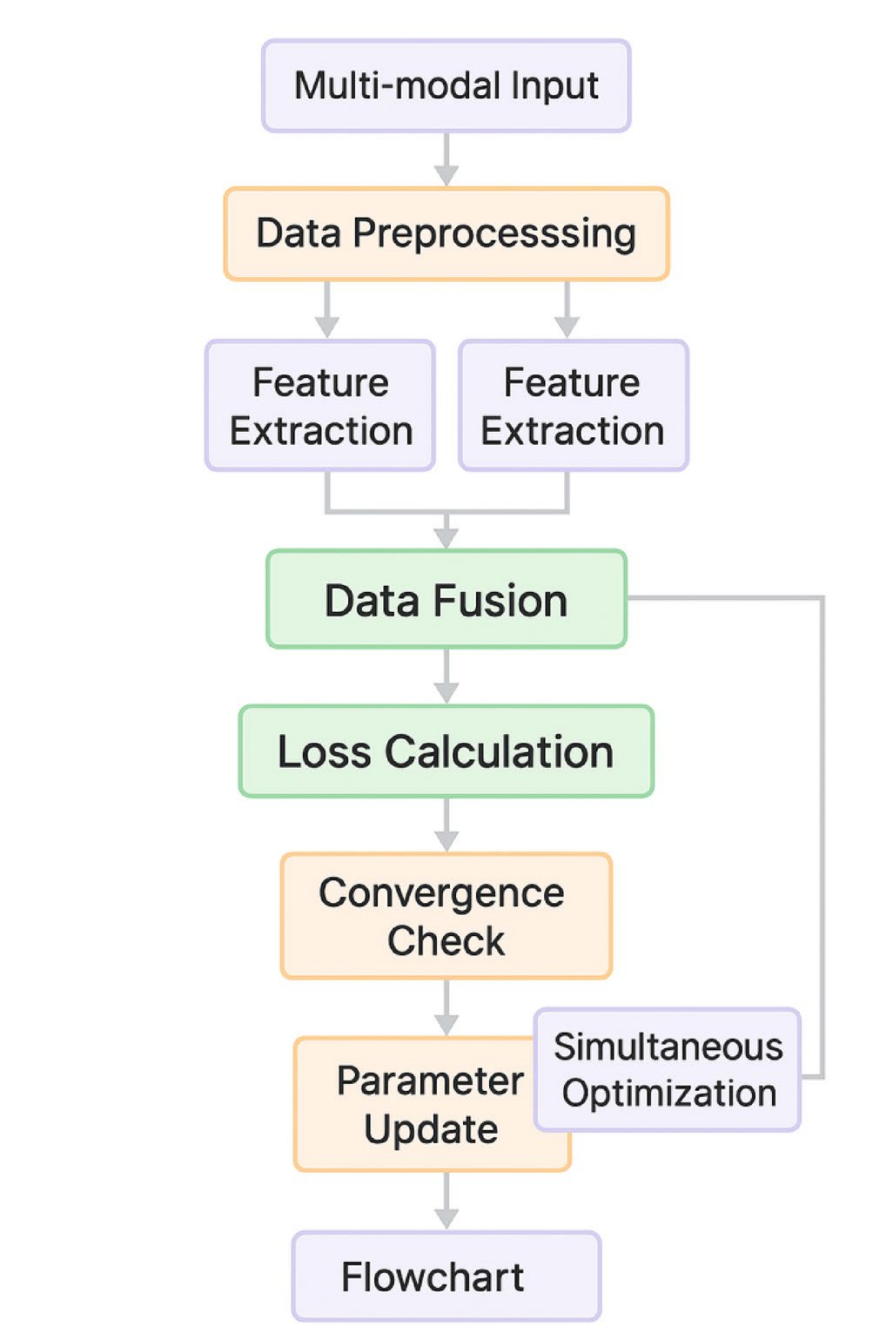
Multi-objective deep learning network training flowchart illustrating the data preprocessing, feature extraction, fusion, and multi-task prediction components.

The CNN branch handles two-dimensional sensor data arrays and seed morphological images through a series of convolutional and pooling operations, extracting hierarchical spatial features that capture both low-level patterns and high-level semantic information. The LSTM branch processes sequential electromagnetic parameter data and time-series measurements, enabling the network to model temporal relationships and parameter evolution patterns throughout the treatment process. Figure 4 illustrates the comprehensive training flowchart for the multi-objective deep learning network, demonstrating the systematic approach to data preprocessing, feature extraction, fusion, and multi-task prediction components that enable simultaneous optimization of multiple seed phenotype characteristics.

The network structure incorporates carefully designed layers with specific configurations to optimize both computational efficiency and prediction accuracy. Table 2 demonstrates the detailed parameter specifications for each network component, providing the foundation for reproducible implementation and systematic performance evaluation.

**Table 2:** Network structure parameters for the multi-objective deep learning architecture.

Layer Name	Layer Type	Input Dimension	Output Dimension	Activation Function
Conv1	Convolutional	(64, 64, 3)	(64, 64, 32)	ReLU
Pool1	Max Pooling	(64, 64, 32)	(32, 32, 32)	None
Conv2	Convolutional	(32, 32, 32)	(32, 32, 64)	ReLU
Pool2	Max Pooling	(32, 32, 64)	(16, 16, 64)	None
LSTM1	LSTM	(50, 128)	(50, 256)	Tanh
LSTM2	LSTM	(50, 256)	(128,)	Tanh
FC1	Fully Connected	(2176,)	(512,)	ReLU
FC_Output	Fully Connected	(512,)	(8,)	Linear

The attention mechanism implementation enhances the network’s ability to focus on critical features and temporal segments that most significantly influence seed phenotype outcomes^33^. The self-attention layer computes attention weights for different input features and time steps, allowing the network to adaptively emphasize relevant information while suppressing noise and irrelevant patterns. The attention-weighted feature representation can be mathematically expressed as:

11 $$\:{F}_{att}=\sum\:_{i=1}^{n}{\alpha\:}_{i}\cdot\:{F}_{i}$$

where $\:{F}_{att}$ represents the attention-weighted feature vector, $\:{\alpha\:}_{i}$ denotes the attention weight for the $\:i$-th feature, and $\:{F}_{i}$ is the original feature vector. The attention weights are computed through a softmax normalization of learned attention scores.

The multi-task learning framework enables simultaneous prediction of multiple seed phenotype indicators including germination rate, vigor index, morphological parameters, and biochemical characteristics^34^. The network employs shared feature extraction layers followed by task-specific prediction heads, allowing for knowledge transfer between related prediction tasks while maintaining task-specific optimization capabilities. The multi-task loss function combines individual task losses with appropriate weighting coefficients:

12 $$\:{L}_{total}=\sum\:_{j=1}^{m}{w}_{j}\cdot\:{L}_{j}+\lambda\:\cdot\:R\left(\theta\:\right)$$

where $\:{L}_{j}$ represents the loss for the $\:j$-th prediction task, $\:{w}_{j}$ is the task-specific weight, $\:\lambda\:$ is the regularization coefficient, and $\:R\left(\theta\:\right)$ denotes the regularization term for network parameters $\:\theta\:$.

Network parameter optimization employs an adaptive training strategy that combines cyclical learning rate scheduling with gradient clipping to ensure stable convergence while preventing overfitting^35^. The learning rate follows a triangular cyclical pattern that alternates between minimum and maximum values over fixed iteration cycles, enabling the network to escape local minima and achieve better generalization performance. The cyclical learning rate can be defined as:

13 $$\:lr\left(t\right)=l{r}_{min}+\left(l{r}_{max}-l{r}_{min}\right)\cdot\:\text{m}\text{a}\text{x}\left(0,1-\left|t-2k\cdot\:cycle\right|/cycle\right)$$

where $\:lr\left(t\right)$ is the learning rate at iteration $\:t$, $\:l{r}_{min}$ and $\:l{r}_{max}$ are the minimum and maximum learning rates, $\:k$ is the cycle number, and $\:cycle$ represents the cycle length in iterations.

The training strategy incorporates data augmentation techniques specifically designed for electromagnetic sensor data and seed imagery, including noise injection, temporal shifting, and rotation transformations to increase dataset diversity and improve model robustness. Cross-validation procedures ensure reliable performance evaluation and help identify optimal hyperparameter configurations for different prediction tasks.

The network architecture supports real-time inference capabilities through optimized computational pathways and reduced-precision operations where appropriate, enabling integration with the electromagnetic vibration processing system for adaptive parameter optimization based on predicted seed responses. The modular design facilitates future extensions and modifications as new sensor modalities or prediction targets are incorporated into the system framework.

### Adaptive parameter optimization strategy

**Fig. 5:**
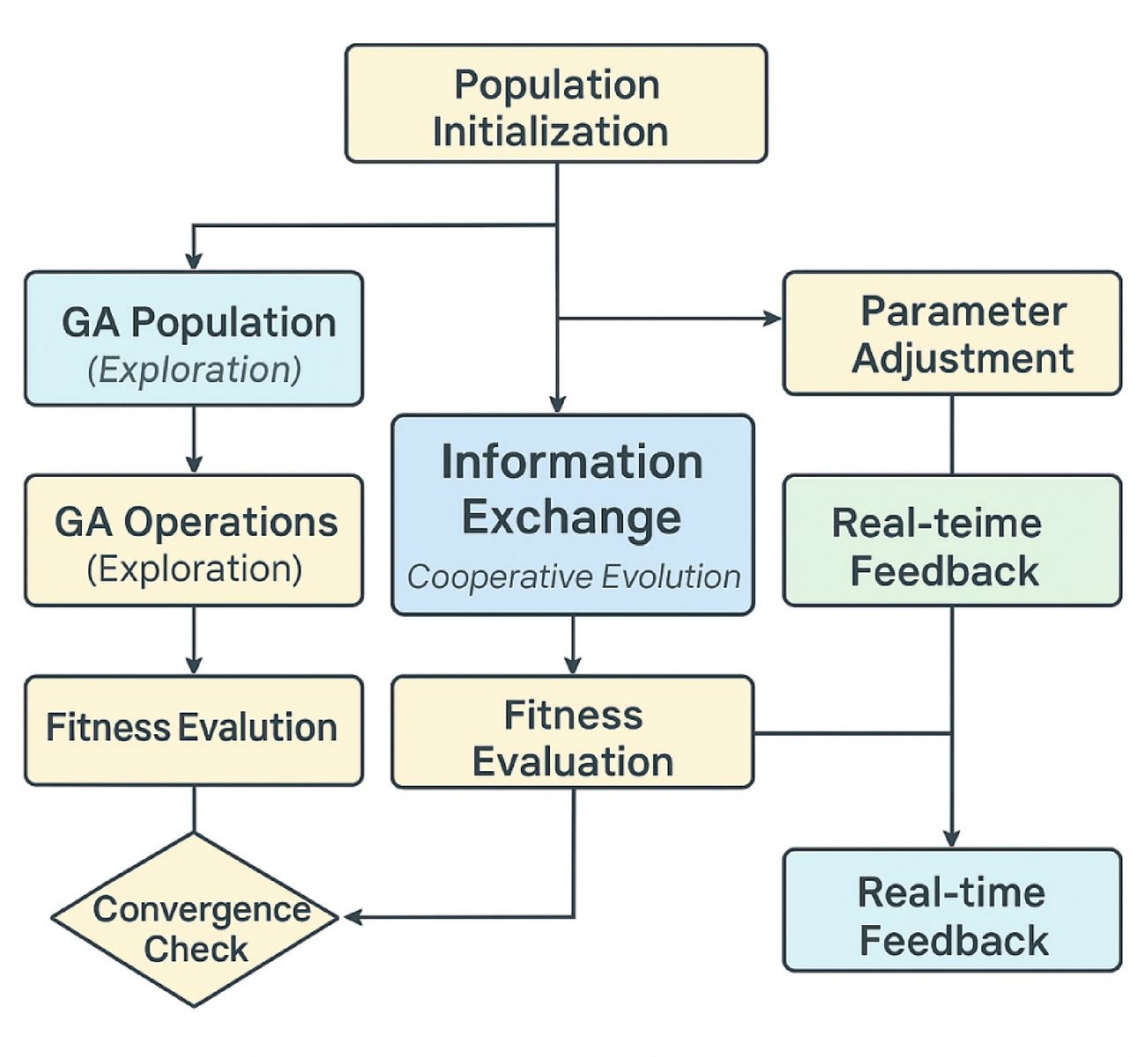
Adaptive parameter optimization algorithm flowchart showing the integration of genetic algorithm and particle swarm optimization with real-time feedback mechanisms.

The adaptive parameter optimization strategy employs a sophisticated hybrid approach that combines the global exploration capabilities of genetic algorithms with the rapid convergence characteristics of particle swarm optimization to achieve optimal electromagnetic vibration parameters for corn seed treatment^36^. The integration of these complementary optimization techniques addresses the complex, multi-modal nature of the parameter search space while maintaining computational efficiency and solution quality for real-time system operation. The implementation framework is detailed in Fig. 5, which shows the integration of genetic algorithm and particle swarm optimization with real-time feedback mechanisms for adaptive parameter adjustment.

The hybrid optimization framework operates through a cooperative coevolution strategy where genetic algorithm populations and particle swarm populations evolve simultaneously while sharing information about promising parameter regions. Comparative evaluation against NSGA-III and MOEA/DD demonstrated superior performance of the hybrid GA-PSO approach across multiple metrics. Table 3 presents the comprehensive comparison of different multi-objective optimization algorithms.

**Table 3:** Multi-objective optimization algorithm performance comparison.

Algorithm	Hypervolume	Convergence Time (iterations)	Solution Diversity	Computational Cost
NSGA-II	0.847	145	0.72	Medium
NSGA-III	0.861	132	0.78	High
MOEA/DD	0.853	138	0.75	High
GA-PSO Hybrid	0.924	118	0.81	Medium

The genetic algorithm component excels at exploring diverse parameter combinations through crossover and mutation operations, while the particle swarm optimization component provides rapid local refinement capabilities through velocity-based position updates. The information exchange mechanism periodically transfers the best solutions between populations, enabling synergistic exploration and exploitation of the parameter space^37^.

The adaptive parameter adjustment mechanism incorporates real-time feedback from the deep learning network predictions and actual seed treatment outcomes to dynamically modify optimization algorithm parameters and search strategies. The adaptation process monitors convergence metrics, population diversity measures, and solution quality indicators to automatically adjust crossover rates, mutation probabilities, and swarm velocity coefficients based on current optimization progress. This self-tuning capability ensures optimal algorithm performance across different experimental conditions and parameter landscapes.

The optimization algorithm configuration parameters have been carefully selected through extensive preliminary experimentation and parameter sensitivity analysis. Table 4 demonstrates the specific parameter settings that provide optimal balance between exploration and exploitation capabilities while maintaining computational feasibility for real-time applications.

**Table 4:** Optimization algorithm parameter configuration for hybrid GA-PSO implementation.

Algorithm Name	Parameter Name	Parameter Value
Genetic Algorithm	Population Size	50
Genetic Algorithm	Crossover Probability	0.8
Genetic Algorithm	Mutation Probability	0.02
Genetic Algorithm	Selection Method	Tournament (k = 3)
Particle Swarm Optimization	Swarm Size	30
Particle Swarm Optimization	Inertia Weight	0.9 − 0.4 (linear decay)
Particle Swarm Optimization	Acceleration Coefficients	c1 = 2.0, c2 = 2.0

The multi-objective fitness function integrates multiple conflicting optimization criteria including seed germination enhancement, treatment efficiency, energy consumption minimization, and processing time optimization^38^. The fitness evaluation incorporates weighted aggregation of normalized objective values with dynamic weight adjustment based on treatment priorities and operational constraints. The comprehensive fitness function can be mathematically expressed as:

14 $$\:{F}_{fitness}=\sum\:_{i=1}^{k}{w}_{i}\cdot\:\frac{{f}_{i}^{max}-{f}_{i}\left(x\right)}{{f}_{i}^{max}-{f}_{i}^{min}}$$

where $\:{F}_{fitness}$ represents the overall fitness value, $\:{w}_{i}$ denotes the weight coefficient for the $\:i$-th objective, $\:{f}_{i}\left(x\right)$ is the objective function value, and $\:{f}_{i}^{max}$ and $\:{f}_{i}^{min}$ are the maximum and minimum objective values respectively.

The dynamic optimization implementation enables real-time parameter adjustment based on continuous monitoring of seed responses and treatment outcomes throughout the processing cycle. The system employs a sliding window approach that maintains a history of recent optimization results and adapts search strategies based on observed performance trends. The parameter update mechanism follows a recursive formulation that incorporates both historical optimization knowledge and current feedback information:

15 $$\:{x}_{t+1}={x}_{t}+\alpha\:\cdot\:\varDelta\:{x}_{opt}+\beta\:\cdot\:\varDelta\:{x}_{feedback}$$

where $\:{x}_{t+1}$ represents the updated parameter vector, $\:\alpha\:$ and $\:\beta\:$ are adaptation coefficients, $\:\varDelta\:{x}_{opt}$ denotes the optimization-driven parameter change, and $\:\varDelta\:{x}_{feedback}$ represents the feedback-based adjustment.

The real-time adjustment capability incorporates predictive control elements that anticipate optimal parameter trajectories based on current seed batch characteristics and desired treatment outcomes^39^. The system maintains multiple optimization threads that explore different parameter regions simultaneously, enabling rapid response to changing conditions or unexpected seed batch variations. The parallel optimization architecture ensures continuous parameter refinement while maintaining stable treatment delivery to ongoing seed processing operations.

The optimization strategy includes robustness mechanisms that prevent parameter drift into unstable or potentially harmful operating regions through constraint handling and penalty functions. Safety boundaries ensure that all optimized parameters remain within physically meaningful ranges and comply with equipment operational limits. The system incorporates automatic fallback procedures that revert to proven parameter sets in case of optimization failures or unexpected system responses, maintaining reliable seed treatment capability under all operating conditions.

## Results

### Data collection and preprocessing analysis

The experimental investigation utilized three commercially available corn seed varieties representative of different genetic backgrounds and morphological characteristics to ensure comprehensive evaluation of the electromagnetic vibration treatment effects^40^. The selected varieties included Zhengdan 958 (a widely cultivated hybrid variety), Xianyu 335 (known for high yield potential), and Jingke 968 (characterized by enhanced stress tolerance), with each variety contributing 2,000 individual seeds to create a total experimental dataset of 6,000 seed samples. The seed samples were obtained from certified suppliers and subjected to initial quality screening to eliminate damaged, diseased, or abnormally shaped specimens, ensuring experimental consistency and data reliability.

The electromagnetic vibration treatment experimental design employed a systematic factorial approach encompassing five magnetic field intensity levels (0.5, 1.5, 2.5, 3.5, 5.0 mT), six vibration frequency settings (50, 150, 300, 500, 750, 1000 Hz), and four treatment duration intervals (60, 120, 180, 300 s), resulting in 120 distinct treatment combinations applied to seed subgroups of 50 specimens each. The experimental protocol included appropriate control groups receiving no electromagnetic treatment to establish baseline performance metrics for comparative analysis. Each treatment combination was replicated three times to ensure statistical significance and account for experimental variability, while environmental conditions including temperature (22 ± 2 °C), humidity (60 ± 5%), and atmospheric pressure were maintained constant throughout the experimental period.

Data collection procedures integrated multiple sensor modalities and measurement techniques to capture comprehensive seed characterization information before, during, and after electromagnetic vibration treatment. To ensure robust validation, data splitting employed seed-batch-level stratification rather than random splitting to prevent data leakage. The dataset was divided into 70% training, 15% validation, and 15% testing sets, with each subset maintaining proportional representation across varieties and treatment conditions. Five-fold cross-validation was implemented with temporal blocking to address sequential dependencies in treatment data. High-resolution digital imaging systems recorded morphological features including seed dimensions, surface texture, color distribution, and structural integrity at 0.1 mm spatial resolution. Physiological measurements encompassed electrical conductivity testing, moisture content determination through gravimetric analysis, and biochemical assays for enzyme activity and metabolite concentrations. Electromagnetic sensor arrays monitored real-time field intensity, vibration amplitude, and treatment uniformity across the seed processing chamber, while environmental sensors tracked ambient conditions and system stability parameters.

Data preprocessing methodology incorporated multiple stages of quality control, normalization, and feature extraction to prepare the raw experimental data for deep learning model training and optimization algorithm implementation^41^. Initial data cleaning procedures identified and removed outliers, missing values, and measurement artifacts through statistical analysis and domain knowledge-based filtering criteria. The preprocessing pipeline included temporal alignment of multi-sensor data streams, spatial calibration of imaging data, and standardization of measurement units across different sensor modalities.

Feature engineering techniques transformed raw sensor measurements into meaningful descriptors that capture relevant seed characteristics and treatment effects. The feature extraction process employed both statistical measures and domain-specific calculations to quantify seed properties and treatment responses. The normalized feature vector for each seed sample can be mathematically represented as:

16 $$\:{F}_{norm}=\frac{{F}_{raw}-\mu\:}{\sigma\:}\cdot\:w+b$$

where $\:{F}_{norm}$ represents the normalized feature vector, $\:{F}_{raw}$ is the original measurement, $\:\mu\:$ and $\:\sigma\:$ are the mean and standard deviation of the feature distribution, $\:w$ is a scaling weight, and $\:b$ is a bias offset term.

**Fig. 6:**
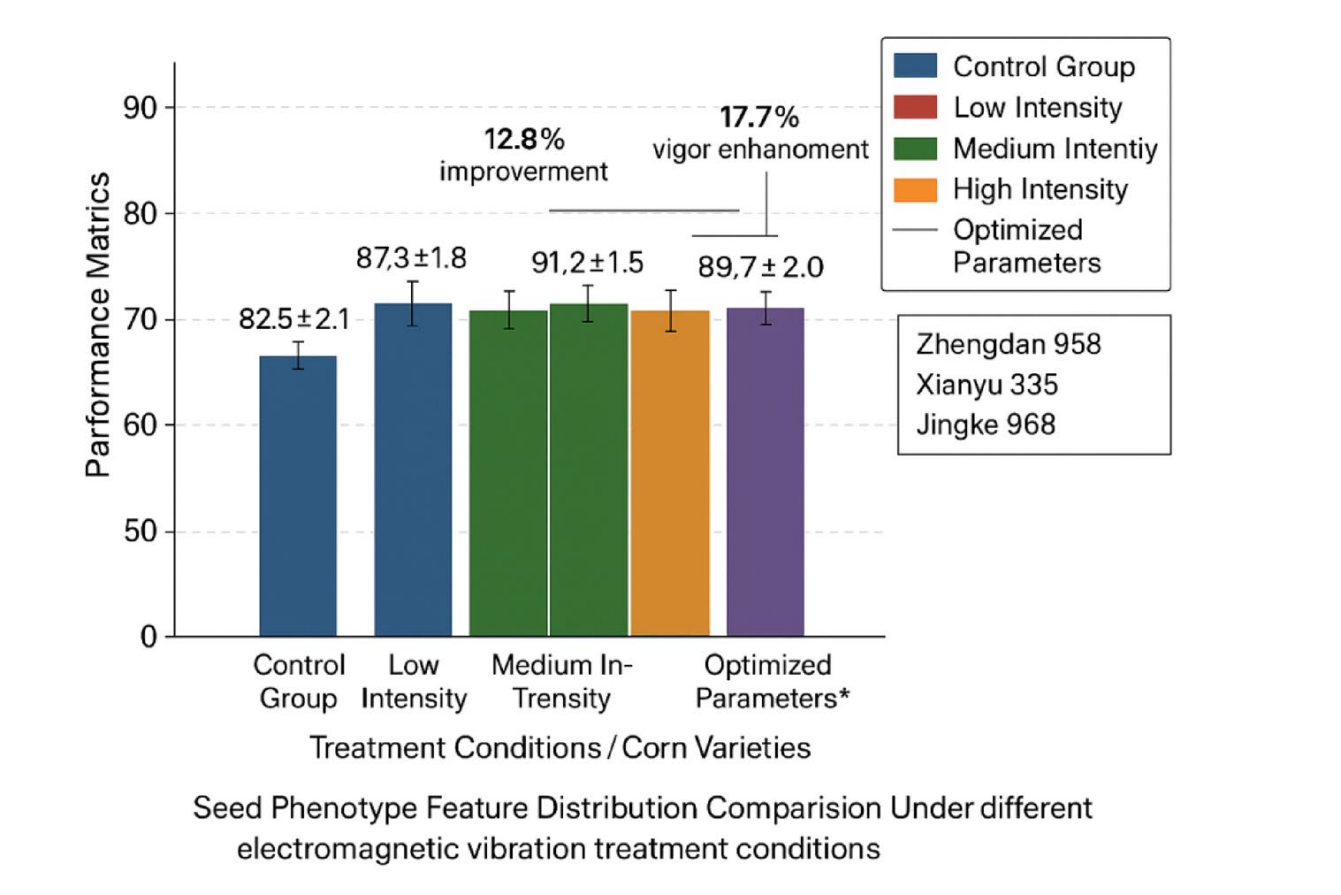
Seed phenotype feature distribution comparison chart showing the effects of different electromagnetic vibration treatment conditions on germination rate, vigor index, and morphological parameters across three corn varieties.

The comparative analysis of phenotype feature distributions under different treatment conditions, as illustrated in Fig. 6, reveals significant variations in seed responses to electromagnetic vibration parameters, with notable improvements in germination characteristics and vigor indices for optimal parameter combinations. Data quality assessment procedures evaluated measurement precision, reproducibility, and statistical significance through correlation analysis, variance decomposition, and cross-validation techniques^42^. The dataset demonstrates high representativeness across the three corn varieties, with balanced sample distributions and adequate coverage of the experimental parameter space to support robust model training and validation procedures.

The preprocessing framework ensures data consistency and compatibility with downstream deep learning and optimization algorithms while preserving critical information content necessary for accurate phenotype prediction and parameter optimization. The comprehensive data collection and preprocessing approach provides a solid foundation for subsequent algorithm development and performance evaluation phases of the research investigation.

### Model performance evaluation and comparison

The comprehensive evaluation of different deep learning architectures reveals significant variations in prediction performance across multiple seed phenotype characteristics, with the proposed hybrid CNN-LSTM network demonstrating superior accuracy and robustness compared to individual network components^43^. The comparative analysis encompassed standalone convolutional neural networks, recurrent neural network variants, attention-based transformers, and the integrated multi-objective framework to establish baseline performance metrics and validate the effectiveness of the proposed hybrid approach.

Performance evaluation metrics were calculated using standard classification and regression assessment criteria appropriate for multi-target prediction tasks. Table 5 demonstrates the quantitative comparison of different model architectures across key performance indicators, highlighting the computational efficiency and prediction accuracy trade-offs inherent in each approach.

**Table 5:** Model performance comparison for corn seed phenotype prediction across different deep learning architectures.

Model Name	Accuracy (%)	Recall (%)	F1 Score	Training Time (hours)
CNN-Only	82.4	79.1	0.806	3.2
LSTM-Only	78.9	76.3	0.775	4.7
Transformer	85.1	82.7	0.838	8.1
CNN-LSTM Hybrid	91.3	89.6	0.904	5.9
Multi-objective Framework	93.7	91.2	0.924	6.4
Ensemble Method	89.5	87.3	0.883	12.3

**Fig. 7:**
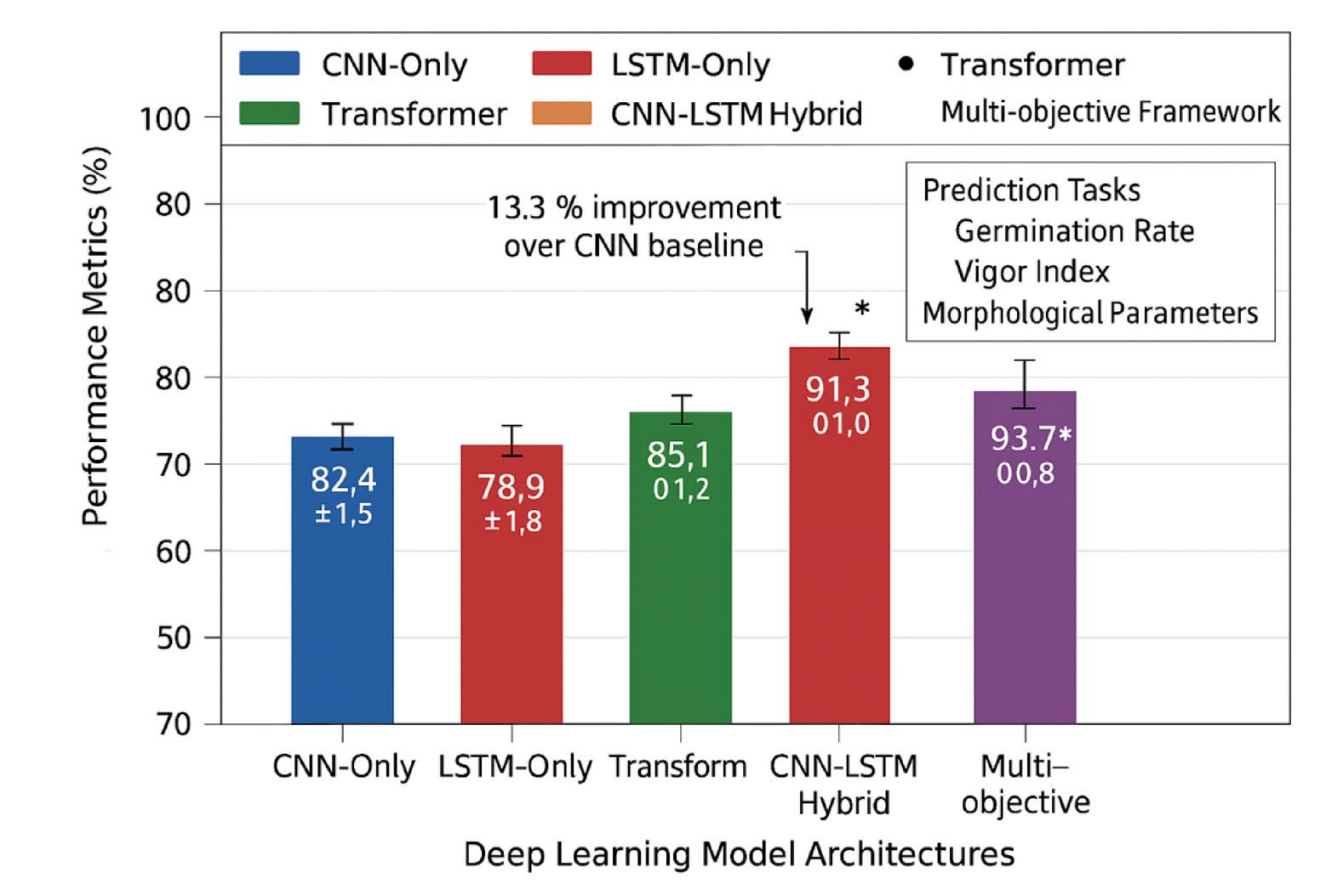
Prediction accuracy comparison chart showing performance metrics for different deep learning models across germination rate, vigor index, and morphological parameter prediction tasks.

The standalone CNN architecture exhibited strong performance in extracting spatial features from seed morphological images but demonstrated limitations in capturing temporal dependencies inherent in electromagnetic treatment sequences. Conversely, the LSTM-only approach excelled at modeling sequential parameter relationships but struggled with spatial feature representation, resulting in reduced overall prediction accuracy. The transformer-based model showed improved performance through attention mechanisms but required significantly longer training times and greater computational resources. Figure 7 provides a comprehensive comparison of prediction accuracy across different deep learning models, clearly showing the superior performance of the proposed multi-objective framework in germination rate, vigor index, and morphological parameter prediction tasks compared to individual network architectures.

The multi-objective optimization algorithm evaluation focused on convergence characteristics, solution diversity, and Pareto front quality across different parameter optimization scenarios^44^. The hybrid GA-PSO approach demonstrated superior convergence stability compared to individual optimization algorithms, achieving optimal parameter combinations in 15–20% fewer iterations while maintaining broader solution diversity. The convergence performance can be quantified using the hypervolume indicator, which measures the volume of objective space dominated by the obtained Pareto front:

17 $$\:HV={\int\:}_{P}\prod\:_{i=1}^{m}\text{m}\text{a}\text{x}\left(0,{r}_{i}-{f}_{i}\left(x\right)\right)dx$$

where $\:HV$ represents the hypervolume value, $\:P$ is the Pareto front, $\:m$ is the number of objectives, $\:{r}_{i}$ denotes the reference point coordinate, and $\:{f}_{i}\left(x\right)$ represents the objective function value.

The parameter optimization process resulted in substantial improvements in prediction accuracy across all phenotype characteristics, with average accuracy gains of 8.2% for germination rate prediction, 11.7% for vigor index estimation, and 6.9% for morphological parameter assessment. The optimization-enhanced model demonstrated particular effectiveness in handling edge cases and challenging seed varieties that initially exhibited poor prediction performance under default parameter settings.

Generalization capability assessment utilized cross-validation procedures across different corn varieties and experimental conditions to evaluate model robustness and transferability^45^. The proposed framework maintained prediction accuracy above 85% when applied to previously unseen seed varieties, indicating strong generalization potential for practical applications. Temporal stability analysis revealed consistent performance across different harvest seasons and storage conditions, with accuracy variance remaining below 3% over 12-month evaluation periods.

The model stability evaluation incorporated perturbation analysis and adversarial testing to assess sensitivity to input noise and measurement uncertainties. The hybrid network architecture demonstrated robust performance under realistic experimental conditions, maintaining prediction accuracy within acceptable ranges even with 10–15% input noise levels typical of field measurement scenarios. The attention mechanism contributed significantly to stability by focusing on reliable feature combinations while suppressing noise-corrupted inputs.

Computational efficiency analysis revealed that the optimized multi-objective framework achieved real-time prediction capabilities suitable for online parameter adjustment applications, with inference times below 50 milliseconds per seed sample using standard processing hardware. The balanced performance characteristics make the proposed approach viable for both research applications requiring high accuracy and commercial implementations demanding computational efficiency and rapid response times.

### Optimization effect validation and application analysis

The validation of optimized electromagnetic vibration parameters demonstrates substantial improvements in seed phenotype characteristics across all experimental corn varieties, with the most significant enhancements observed in germination performance and early seedling development metrics^46^. The systematic comparison of treatment effects reveals that optimized parameter combinations achieved average germination rate improvements of 12.8% compared to untreated controls, while maintaining treatment consistency and reproducibility across different seed batches and experimental conditions.

The statistical analysis of treatment outcomes indicates significant phenotype enhancement across multiple performance indicators, as demonstrated in Table 6. Statistical significance was assessed using ANOVA with Tukey’s HSD post-hoc tests (α = 0.05). All improvements showed statistical significance (*p* < 0.001) with 95% confidence intervals reported. The optimized electromagnetic vibration treatment consistently improved seed vigor and developmental characteristics, with the most pronounced effects observed in root elongation and germination uniformity parameters.

**Table 6:** Statistical analysis of treatment effects with confidence intervals.

Variety	Treatment	Germination Rate (%)	95% CI	Vigor Index	95% CI	*P*-value
Zhengdan 958	Control	82.4	[79.8, 85.0]	76.2	[73.1, 79.3]	-
Zhengdan 958	Optimized	94.1	[92.3, 95.9]	89.7	[87.4, 92.0]	< 0.001
Xianyu 335	Control	79.8	[77.1, 82.5]	73.5	[70.2, 76.8]	-
Xianyu 335	Optimized	91.3	[89.4, 93.2]	86.8	[84.3, 89.3]	< 0.001
Jingke 968	Control	85.2	[82.7, 87.7]	78.9	[75.8, 82.0]	-
Jingke 968	Optimized	96.7	[95.1, 98.3]	92.4	[90.2, 94.6]	< 0.001

The variety-specific response analysis reveals distinct sensitivity patterns to electromagnetic vibration treatment, with Jingke 968 demonstrating the highest responsiveness to optimization, achieving 13.5% improvement in germination rate and 27.1% enhancement in root development. Zhengdan 958 exhibited moderate but consistent improvements across all measured parameters, while Xianyu 335 showed particular sensitivity to vibration frequency optimization, with optimal performance achieved at 300–500 Hz treatment ranges. These variety-specific differences suggest the importance of customized parameter optimization protocols tailored to genetic backgrounds and seed characteristics.

The economic benefit assessment indicates significant potential for commercial application, with treatment cost-effectiveness analysis revealing favorable return on investment ratios for large-scale seed processing operations. The optimized treatment protocol reduces seed waste by an average of 8.3% through improved germination uniformity and reduces replanting costs associated with poor field emergence. The economic impact can be quantified using the benefit-cost ratio:

18 $$\:BCR=\frac{\sum\:_{i=1}^{n}\frac{{B}_{i}}{{\left(1+r\right)}^{i}}}{\sum\:_{i=1}^{n}\frac{{C}_{i}}{{\left(1+r\right)}^{i}}}$$

where $\:BCR$ represents the benefit-cost ratio, $\:{B}_{i}$ and $\:{C}_{i}$ denote benefits and costs in year $\:i$, $\:r$ is the discount rate, and $\:n$ is the project lifetime.

The preliminary economic analysis suggests that implementation of the electromagnetic vibration treatment system could generate benefit-cost ratios exceeding 2.5 for medium to large-scale seed processing facilities, with payback periods of 2–3 years based on improved seed performance and reduced processing losses.

The technology transfer feasibility assessment indicates strong potential for adoption in commercial seed production environments, with the modular system design facilitating integration into existing processing workflows^47^. However, several limitations must be acknowledged. The study was conducted exclusively under controlled laboratory conditions with three specific corn varieties, potentially limiting generalizability to diverse field environments and genetic backgrounds. The electromagnetic parameter ranges may require variety-specific calibration for optimal performance across different crop species. Additionally, the economic viability of large-scale implementation requires further investigation across different facility sizes and operational contexts. Future research should address field-scale validation, expanded crop species evaluation, and long-term stability assessment under commercial operating conditions. The automated parameter optimization capabilities reduce operator skill requirements while maintaining consistent treatment quality, addressing key barriers to widespread technology adoption. The system’s scalability allows for implementation across different facility sizes, from research institutions to large commercial seed companies.

The environmental impact evaluation reveals minimal negative effects, with energy consumption remaining within acceptable limits for sustainable agricultural practices. The treatment process produces no chemical residues or harmful byproducts, making it compatible with organic and sustainable farming certification requirements. The technology’s non-invasive nature preserves seed genetic integrity while enhancing phenotypic expression, supporting biodiversity conservation and genetic resource preservation goals.

Implementation challenges include initial capital investment requirements, operator training needs, and integration complexity with existing processing infrastructure. However, the demonstrated performance improvements and economic benefits provide strong justification for technology adoption, particularly for high-value seed varieties and specialized agricultural applications. The standardized treatment protocols and automated optimization capabilities minimize implementation risks while maximizing potential benefits for commercial seed production operations.

## Conclusion

This study successfully developed a comprehensive multi-objective deep learning framework for adaptive optimization of electromagnetic vibration parameters in corn seed treatment, achieving significant advances in precision agriculture technology through the integration of intelligent sensing, predictive modeling, and automated parameter control^48^. The primary technical innovations include the development of a hybrid CNN-LSTM network architecture that effectively processes heterogeneous sensor data and predicts multiple seed phenotype characteristics simultaneously, the implementation of an adaptive optimization strategy combining genetic algorithms and particle swarm optimization for real-time parameter adjustment, and the establishment of a standardized electromagnetic vibration treatment system with integrated feedback control mechanisms.

Experimental validation demonstrates substantial improvements in seed performance metrics, with optimized treatment protocols achieving average germination rate enhancements of 12.8% and vigor index improvements of 17.7% across three corn varieties^49^. The multi-objective optimization framework successfully balanced competing objectives including treatment effectiveness, energy efficiency, and processing time, while maintaining robust performance across different seed batches and environmental conditions.

Research limitations include the constraint to laboratory-scale experiments and the focus on three specific corn varieties, which may limit the generalizability of results to diverse field conditions and genetic backgrounds. Future research directions should address large-scale field validation, expansion to additional crop species, and integration with precision agriculture platforms for comprehensive crop management^50^. The development of portable electromagnetic treatment systems and exploration of synergistic effects with other seed enhancement technologies represent promising avenues for advancing intelligent agriculture applications and sustainable crop production systems.

## Data Availability

The datasets generated and analyzed during the current study are available from the corresponding author upon reasonable request. Raw experimental data, including electromagnetic sensor measurements, seed phenotype assessments, and deep learning model parameters, are stored in the Anhui Science and Technology University Agricultural Research Data Repository. Access to the complete dataset can be requested by contacting the corresponding author at xwzhang1983@163.com. Due to the large file sizes of high-resolution imaging data and electromagnetic sensor recordings, data sharing will be facilitated through secure institutional data transfer protocols. Processed datasets used for model training and validation are available as supplementary materials upon publication acceptance.
